# A study of antioxidant activity in patients with schizophrenia taking atypical antipsychotics

**DOI:** 10.1007/s00213-014-3624-0

**Published:** 2014-05-29

**Authors:** Marilena Gilca, Gabriela Piriu, Laura Gaman, Corina Delia, Liviu Iosif, Valeriu Atanasiu, Irina Stoian

**Affiliations:** 1 Department of Biochemistry, UMF Carol Davila, Bucharest, Romania; 2 SC R&D IRIST LABMED SRL, Bucharest, Romania; 3 Department of Psychiatry, Sapoca Psychiatric Hospital, Buzău, Romania; 4 Department of Biochemistry, Alfred Rusescu Institute for Mother and Child Care, Bucharest, Romania

**Keywords:** PON, TEAC, Antioxidant, Atypical antipsychotic, Arylesterase

## Abstract

**Introduction:**

Atypical antipsychotics have significantly improved the quality of life for schizophrenic patients. Despite their beneficial effects, these antipsychotics induce weight gain, diabetes, and dyslipidemia. The aims of this study were to investigate the antioxidative activity of paraoxonase and assess lipid profile as a cardiovascular risk factor in patients with schizophrenia under long-term clozapine or risperidone treatment.

**Methods:**

The study included 66 patients with schizophrenia under clozapine or risperidone treatment and 19 healthy control subjects. Serum paraoxonase activities against paraoxon (PON(PO)), phenylacetate (PON(PA)), dihydrocoumarin (PON(DHC)), serum Trolox equivalent antioxidant activity (TEAC), antioxidant gap (GAP), and lipid profile were determined.

**Results:**

PON(DHC) activity was reduced in both antipsychotic drug-treated groups (clozapine 43.46 ± 1.06 U/ml, *p* < 0.001; risperidone 50.57 ± 1.54 U/ml, *p* < 0.01; control 52.27 ± 1.34 U/ml). A similar pattern was observed for the PON(DHC)/HDL-cholesterol (HDLC) ratio. On the contrary, PON(PO) and PON(PA) were increased in the treated group, but the corresponding paraoxonase/HDLC ratios were not significantly different from controls, except for PON/HDLC in the clozapine group. TEAC and GAP were only decreased in the clozapine-treated group.

**Conclusions:**

In patients with schizophrenia, clozapine or risperidone treatment had different effects on various paraoxonase activities. The results of the present study suggest that patients with schizophrenia might be at increased risk for metabolic and cardiovascular disease related to reduced PON(DHC), TEAC, and GAP.

## Introduction

Atypical antipsychotics introduced after 1990 have significantly reduced the frequency of acute extrapyramidal symptoms and improved the quality of life for patients with schizophrenia. Unfortunately, substantial weight gain, glucose dysregulation, and hyperlipidemia induced by these drugs are important concerns for many individuals because these adverse effects are more common and severe with atypical antipsychotics than with conventional ones (Allison et al. 1999a; Ruetsch et al. 2005). Moreover, levels of mortality from obesity-related conditions, such as coronary heart disease, are higher in patients with schizophrenia (Allison et al. 2009).

Among the atypical antipsychotics agents, clozapine and olanzapine appear to have the greatest potential to induce weight gain, diabetes, and dsylipidemia, while risperidone has an intermediate effect (Allison et al. 1999b; Baptista et al. 2008).

Paraoxonases are relatively newly identified antioxidant enzymes that are synthesized by the liver and transported almost exclusively on high-density lipoprotein (HDL). Paraoxonase 1 (PON1) has been the focus of recent cardiovascular research because of its evident capacities to protect low-density lipoproteins (LDLs) against oxidative stress and prevent atherogenesis (Precourt et al. 2011). These protective functions of PON1 may be related to its ability to hydrolyze oxidized lipids on LDL and to prevent the accumulation of oxidized LDL, which is believed to be central to the initiation and progression of atherosclerosis (Mackness et al. 2003). Previous studies have shown that individuals with low PON activity, regardless of genotype, are at greater risk for developing cardiovascular disease (CAD) (Durrington et al. 2001; Mackness et al. 2001; Jarvik et al. 2000). Nevertheless, the influence of atypical antipsychotic drugs on PON activity and its relationship with metabolic and cardiovascular risk factors in psychiatric patients remain to be fully elucidated. Only two studies, having partially contradictory results, have evaluated the impact of atypical antipsychotic drugs on PON activity. The first study found no significant difference between PON activity levels in female patients receiving antipsychotics and those in controls (Ozenoglu et al. 2008). Despite this, PON was positively correlated with body mass index (BMI), and the PON1/HDL ratio was positively correlated with triglyceride levels in subjects under treatment, but not in controls. The second study found reduced serum PON1 activity in patients with schizophrenia treated with olanzapine but not quetiapine, when compared with controls, while serum levels of total cholesterol and LDL-C in the olanzapine group were significantly higher than those of quetiapine and control groups (Ünsal et al. 2013). These results, although partially contradictory, suggest a potential contribution of paraoxonase level and/or activity changes in atypical antipsychotic drug-induced cardiovascular risk. Nevertheless, the impact of atypical antipsychotic drugs on atherogenesis remains unclear. Several studies have shown that long-term treatment with atypical antipsychotics may induce an oxidant/antioxidant imbalance and increase lipid peroxidation (Fehsel et al. 2005; Gama et al. 2006; Zhang et al. 2006). Levels of superoxide dismutase (SOD) were significantly increased in patients with schizophrenia (Zhang et al. 2006, 2012), but risperidone treatment reduced elevated blood SOD levels in schizophrenic subjects (Zhang et al. 2012). Clozapine-induced protein oxidation has been suggested as a possible mechanism of antipsychotic-associated metabolic alterations (Baig et al. 2010; Walss-Bass et al. 2008).

The purpose of our study was therefore to investigate the impact of long-term atypical antipsychotic treatment with clozapine and risperidone on cardiovascular risk and antioxidant protection markers, including serum paraoxonase activities against paraoxon (both basal and NaCl-stimulated PON activity), phenylacetate (arylesterase PON activity), and dihydrocoumarin (lactonase PON activity). Because lactonase PON activity is hypothesized to be responsible for the antioxidant capacity of HDL (Gaidukov and Tawfik 2007), we also measured plasma total antioxidant activity (TEAC) and antioxidant gap (GAP).

## Material and methods

### Subjects

The present study was an observational study with cross-sectional design. The subjects were selected from outpatients treated at a psychiatric hospital in Sapoca, Buzău, Romania, between December 2010 and June 2011. A senior psychiatrist identified patients who met the following criteria: (1) fit the Diagnostic and Statistical Manual of Mental Disorders IV (DSM-IV) criteria of schizophrenia (First and Pincus 1999), (2) a minimum of 3 years’ duration of disease, and (3) a 1-year minimum duration of antipsychotic treatment with clozapine or risperidone; these subjects were asked to participate in the study. All patients were evaluated with the Item Group Checklist section of the Schedules for Clinical Assessment in Neuropsychiatry (SCAN) in order to confirm the diagnosis (Wing et al. 1990). The exclusion criteria were the presence of acute or chronic illnesses known to affect the immune, endocrine, or metabolic systems, and any additional chronic medications. The study was approved by the local ethics committee and conducted according to the ethical obligations of the Declaration of Helsinki. From the initial sample of 70 patients, 4 were excluded for physical reasons (3 patients with acquired immune deficiency syndrome [AIDS] and 1 with hepatitis C), leaving 66 patients (44 under clozapine treatment [10 females and 34 males; 27 nonsmokers and 15 smokers], and 22 under risperidone treatment [7 females and 15 males; 15 nonsmokers and 5 smokers]) who were entered into the study. We have selected clozapine and risperidone for our study, since these two drugs are already known among the atypical antipsychotic drugs as potent (clozapine) or intermediate (risperidone) inducers of lipid metabolism alterations (Allison et al. 1999b; Baptista et al. 2008). Taking into account their proved dyslipidemic potential, we intended to compare also their strength in terms of the oxidative capacity. We also enrolled 19 healthy subjects (12 females and 7 males; 9 nonsmokers and 5 smokers) without current or past psychiatric disorders as controls, using the same exclusion criterion described above. Neither patients nor controls had alimentary restrictions, and no vegetarian or vegan subjects were included in the study. There was no statistical trend difference among the groups, in terms of smoker/nonsmoker distribution (chi-square test). After a complete description of the study, all patients and healthy controls provided informed consent to participate in the investigation. All of the subjects in both groups were of European Caucasian origin.

The average durations of illness were 11.4 ± 5.6 and 9.4 ± 4.7 years, and treatment durations were was 5.8 ± 4.7 and 6.1 ± 5.4 years in the clozapine and risperidone groups, respectively. Clozapine dosages ranged from 100 to 450 mg daily throughout the treatment period, while risperidone dosing ranged from 2 to 6 mg daily. None of the subjects experienced agranulocytosis during the study period.

### Sample processing

All subjects underwent blood sampling (10 ml) into heparin-containing tubes after an overnight fast. After centrifugation, the plasma was retained on ice for PON activity and TEAC assays. Reagents and ultrapure water were treated with Chelex 100 (Merck, Darmstadt, Germany) to bind transitional metals. All reagents were of pure analytical quality and were purchased from Sigma-Aldrich Chemie (Steinheim, Germany), unless otherwise indicated. All assays were carried out on duplicate samples on a Perkin-Elmer Lambda EZ 210 UV–VIS spectrophotometer (Perkin-Elmer Inc., Boston, MA, USA) or on a Cobas Mira Plus automatic analyzer (Roche Diagnostics, Basel, Switzerland). Serum PON enzymatic activity was spectrophotometrically determined using three different substrates.

### PON activity against paraoxon (PON(PO))

To measure paraoxonase activity, serum was incubated in Tris–HCl buffer (100 mmol/l, pH 8.0) containing 5.5 mmol/l paraoxon (*O,O-*diethyl *O-p-*nitrophenyl phosphate, Sigma-Aldrich Chemie) and 2 mmol/l CaCl_2_ either with 1 mol/l NaCl (salt-stimulated activity: PON(PO-NaCl)) or without NaCl (basal activity: PON(PO)). The generation rate of the product, *p-*nitrophenol, was monitored at 412 nm. Enzyme activity was calculated from its molar extinction coefficient 18,290 M^−1^ cm^−1^. One unit of PON is defined as 1 nmol *p-*nitrophenol/ml/min under the above-described assay conditions (Richter et al. 2004).

### Arylesterase PON activity (PON(PA))

To measure arylesterase activity, serum was added to Tris–HCl buffer (100 mmol/l, pH 8.0) containing 2 mmol/l CaCl_2_ and 2 mmol/l phenylacetate (acetic acid phenyl ester 99 %; Sigma-Aldrich Chemie). The rate of phenylacetate hydrolysis was monitored at 270 nm. After subtracting the nonenzymatic hydrolysis, enzyme activity was calculated from the molar extinction coefficient of the product, 1.310 M^−1^ cm^−1^. One unit of PON(PA) activity is defined as 1 μmol of *p-*nitrophenol/ml/min under the above-described assay conditions (Haagen and Brock 1992; Kawai et al. 1990).

### Lactonase PON activity (PON(DHC))

Lactonase PON activity was measured using dihydrocoumarin (DHC) as substrate. Briefly, serum and substrate were added in the buffer, and the absorbance was monitored at 270 nm. Activities are expressed as units per milliliter (Gaidukov and Tawfik 2007).

### Plasma total antioxidant activity (TEAC)

Plasma total antioxidant activity was determined based on the 6-hydroxy-2,5,7,8-tetramethylchroman-2 carboxylic acid (Trolox) equivalent antioxidant capacity assay developed by Miller et al. with modifications (Miller and Rice-Evans 1996; Re et al. 1999). The TEAC assay measures the relative abilities of antioxidants to scavenge the 2,2′-azino-bis (3-ethylbenzothiazoline-6-sulfonic acid) (ABTS) radical cation (ABTS^*+^) compared with the antioxidant potency of standard amounts of Trolox, the water-soluble vitamin E analog. The ABTS radical was generated from the interaction between ABTS and potassium persulfate. Solution containing ABTS^*+^ was added to the serum samples, and the absorbance was read after 1 min at 734 nm and compared to that of 5 mM phosphate buffer. We calculated the percentage inhibition of the absorbance, which is directly proportional to the antioxidant activity of the sample. The assay was calibrated against a calibration curve with Trolox as the standard, and the results are expressed as millimoles per liter of Trolox.

### Plasma residual antioxidant activity (“antioxidant gap”; GAP)

The principal antioxidants (by mass and activity) of human plasma are albumin and uric acid, which account for 51–57 % of the total antioxidant activity (Miller and Rice-Evans 1996; Miller et al. 1997). Antioxidant gap reflects the combined activity of other extracellular antioxidants and was calculated by subtracting the antioxidant activity ascribable to albumin and uric acid from the TEAC value for each sample according to the formula: GAP = TEAC − [(Albumin × 0.69) + uric acid], where 0.69 is the TEAC value for human serum albumin, while 1.0 is the TEAC value for serum uric acid; albumin = serum albumin concentration (expressed as mmol/L); uric acid = serum uric acid concentration (expressed as mmol/L). The results were expressed as millimoles per liter of Trolox activity.

### Routine biochemical analyses

We quantified total cholesterol (TC), triglyceride (TG), HDL-cholesterol (HDLC), albumin, and uric acid levels using commercially available kits from DiaSys (Holzheim, Germany).

### Statistical analysis

All data are presented as adjusted predictive values based on a linear regression model using age and BMI as independent variables. No data imputations were performed. The data were therefore corrected for age and BMI as potential confounders. Data analysis was performed using GraphPad InStat software package (GraphPad Software, Inc., La Jolla, CA, USA). Differences between groups were computed using analysis of variance (ANOVA) with parametric (Tukey’s) or nonparametric (Kruskal–Wallis) post hoc tests. The strength of association between pairs of variables was assessed by Pearson’s correlation coefficient. A *p* value <0.05 was considered statistically significant.

## Results

A total of 44 clozapine-treated and 22 risperidone-treated patients were enrolled in the study. All patients fulfilled the DSM-IV criteria for schizophrenia. The participants’ demographic and clinical characteristics are shown in Table 1.

**Table 1:** Clinical and demographic characteristics of the patients and controls

	Schizophrenia subjects—clozapine group (*N* = 44)	Schizophrenia subjects—risperidone group (*N* = 22)	Control group (*N* = 19)	*p* values—clozapine vs. control	*p* values—risperidone vs. control	*p* values—clozapine vs. risperidone
Age (years)	38.47 (1.50)	47.47 (3.19)	62.31 (11.67)	<0.001	<0.001	<0.05
BMI (kg/m^2^)	27.145 (0.82)	23.71 (0.59)	23.59 (1.88)	<0.05	n.s.	<0.05
TG^a^ (mg/dl)	182.64 (14.36)	140.47 (3.05)	140.47 (3.05)	<0.001	n.s.	<0.05
CT^b^ (mg/dl)	275.82 (11.35)	237.47 (1.67)	230.82 (2.26)	<0.001	n.s.	<0.05
HDLC^c^ (mg/dl)	48.20 (2.64)	41.30 (0.87)	37.168 (0.54)	<0.001	<0.01	<0.001
Albumin (mg/dl)	5.80 (0.13)	5.81 (0.21)	5.17 (0.06)	<0.05	<0.05	n.s.
Acid uric (mg/dl)	5.14 (0.42)	4.64 (0.51)	4.92 (0.23)	n.s.	n.s.	n.s.

All the values are expressed as mean (SEM)

^a^For TG, *N* = 43 for the clozapine group, *N* = 21 for the risperidone group, and *N* = 15 for the control group

^b^For CT, *N* = 43 for the clozapine group, *N* = 22 for the risperidone group, and *N* = 16 for the control subjects

^c^For HDLC, *N* = 43 for the clozapine group, *N* = 21 for risperidone group, and *N* = 17 for the control subjects

Clozapine-treated patients had significantly lower age (*p* < 0.001) and higher BMI (*p* < 0.05), TG (*p* < 0.001), CT (*p* < 0.001), and HDLC values (*p* < 0.001) than control subjects, while risperidone-treated subjects were younger (*p* < 0.001) but did not differ from the control group with regard to BMI, TG, or CT (Table 1). The two groups of schizophrenic patients also differed significantly in all these characteristics (Table 1).

**Fig. 1:**
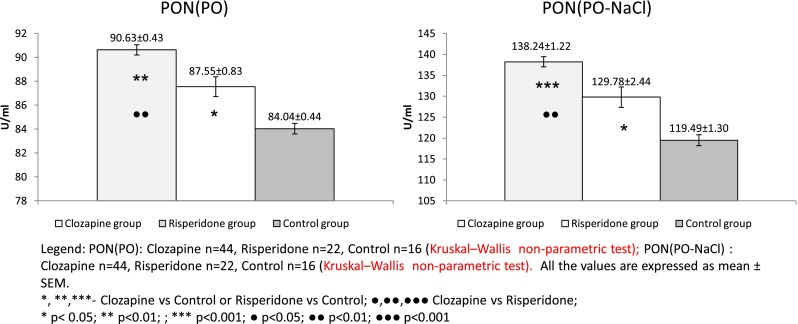
PON(PO) and PON(PO-NaCl) values in clozapine- and risperidone-treated schizophrenic patients vs. the control group

**Fig. 2:**
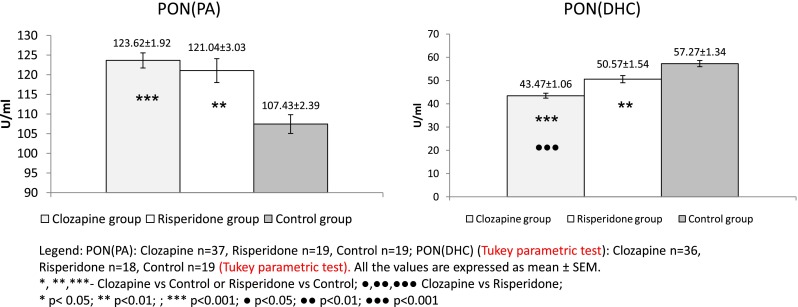
PON(PA) and PON(DHC) values in clozapine- and risperidone-treated schizophrenic patients vs. the control group

**Fig. 3:**
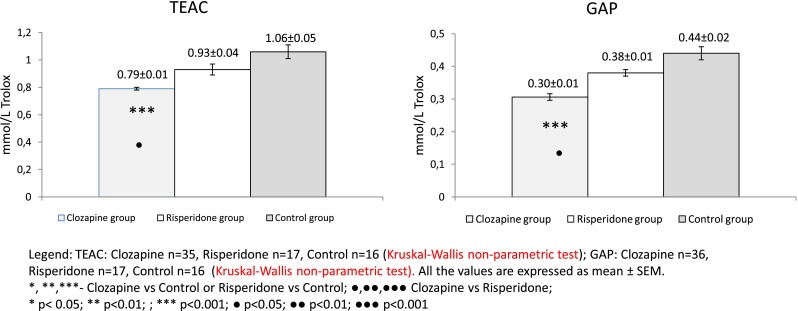
TEAC and GAP values in clozapine- and risperidone-treated schizophrenic patients vs. the control group

Data regarding oxidative stress-related parameters are shown in Figs. 1, 2, and 3. Paraoxonase activities against paraoxon as substrate (either basal or stimulated activity) were significantly higher in both groups of patients with schizophrenia than in control subjects (Fig. 1). The same variation was observed for PON(PA) (Fig. 2). Conversely, PON(DHC) was significantly lower in the two groups of patients than in the controls (Fig. 2).

Plasma HDLC concentrations were significantly higher in both groups of patients than in control subjects (Table 1). To assess whether altered paraoxonase activity was due to a change in HDL level or to a stabilizing effect of HDL on paraoxonase, we calculated paraoxonase/HDLC ratios (Table 2).

**Table 2:** PON/HDLC ratios

	Schizophrenia subjects treated with clozapine	Schizophrenia subjects treated with risperidone	Control subjects	*p* values—clozapine vs. control	*p* values—risperidone vs. control	*p* values—clozapine vs. risperidone
PON(PO)/HDLC ratio (U/mg)	202.20 (1.86) (*N* = 42)	213.30 (2.48) (*N* = 20)	222.45 (1.72) (*N* = 14)	<0.001	n.s.	<0.01
PON(PO-NaCl)/HDLC ratio (U/mg)	308.77 (2.87) (*N* = 42)	298.65 (14.99) (*N* = 21)	315.22 (1.18) (*N* = 14)	n.s.	n.s.	n.s.
PON(PA)/HDLC ratio (U/mg)	274.39 (5.36) (*N* = 36)	288.28 (4.61) (*N* = 17)	288.48 (3.91) (*N* = 11)	n.s.	n.s.	n.s.
PON(DHC)/HDLC ratio (U/mg)	98.55 (3.80) (*N* = 36)	127.76 (6.13) (*N* = 16)	160.60 (7.32) (*N* = 12)	<0.001	<0.01	<0.001

All the values are expressed as mean (SEM)

We found that the PON(DHC)/HDLC ratio was significantly lower in the clozapine (*p* < 0.001) and risperidone (*p* < 0.01) groups than controls. The variation was significantly higher in the clozapine group (*p* < 0.001) than in the risperidone group. Moreover, while PON(PO) activity increased after treatment with both atypical antipsychotic drugs, the PON(PO)/HDLC ratio was significantly decreased in the clozapine group (*p* < 0.01). Although there was no difference between the risperidone and control groups in terms of the PON(PO)/HDLC ratio, there was a significant difference between the clozapine and risperidone groups (*p* < 0.01). PON(PO-NaCl)/HDLC and PON(PA)/HDLC were not significantly different among the three groups. TEAC and GAP were significantly lower in the clozapine group than in the control group (both *p* < 0.001, Fig. 3).

Although there was no difference between the risperidone and control groups in terms of TEAC or GAP, there was a significant difference between the clozapine and risperidone groups (*p* < 0.01 and *p* < 0.05, respectively). We also found that PON(DHC) was positively correlated with TEAC and GAP in all three groups (clozapine: TEAC *r* = 0.478, *p* < 0.01, GAP *r* = 0.775, *p* < 0.0001; risperidone: TEAC *r* = 0.927, *p* < 0.0001, GAP *r* = 0.985, *p* < 0.0001; control: TEAC *r* = 0.973, *p* < 0.0001, GAP *r* = 0.994, *p* < 0.0001). We found no sex-associated differences in any of the oxidative stress-related parameters, either in schizophrenia or control groups.

## Discussion

An increasing amount of evidence suggests a possible increase in cardiovascular events in patients with schizophrenia treated with atypical antipsychotic drugs, and this is hypothesized to be secondary to lipid (Allison et al. 1999a, b, 2009; Ruetsch et al. 2005), as well as to glucose metabolism dysregulation (Henderson et al. 2005; Zhang et al. 2014). Serum paraoxonase is a HDL-associated hydrolase with a broad range of substrates (paraoxon, phenylacetate, dihydrocoumarin) and inhibits LDL oxidation. Previous studies have shown that paraoxonase activity towards paraoxon is inversely related to the level of CAD and represents a predictive risk factor for subsequent coronary events independent of all other established risk factors, with the exception of HDL (Ayub et al. 1999; Mackness et al. 2003). The significant decreases in PON(DHC) activities in clozapine- and risperidone-treated patients in the present study support the hypothesis of high cardiovascular risk associated with chronic atypical antipsychotic drug administration. Although we found increased activities of PON(PO) and PON(PA), it is important to emphasize that lactonase activity is the endogenous activity of paraoxonase and directly mediates its anti-atherogenic functions. Whereas traditional paraoxonase and arylesterase assays weakly reflect paraoxonase-HDL complex levels, lactonase activity correlates significantly better with the degree of paraoxonase binding to HDL and may thus provide a better indication of the antioxidant and anti-atherogenic capacities of the HDL particles on which the enzyme is anchored (Gaidukov and Tawfik 2007). Therefore, we also calculated the paraoxonase/HDLC ratios (see Table 2).

Surprisingly, PON/HDLC was significantly lower in the clozapine group than in either the control group (*p* < 0.001) or the risperidone group (*p* < 0.01). It is already established that a decreased paraoxonase/HDL ratio may lead to a reduction in the antioxidant capacity of HDL, which might contribute to the accelerated development of atherosclerosis (Paragh et al. 1999). Thus, our results suggest that paraoxonase/HDL may be a better predictor of atherosclerosis in patients with schizophrenia treated with clozapine or risperidone than plasma HDLC concentrations or paraoxonase activity. The present study also demonstrates that HDLC increases in patients with schizophrenia who are treated with atypical antipsychotic drugs, suggesting that HDLC may not be a strict requirement for antioxidant or even cardiovascular protection. Also another study showed that the cardiovascular predictive value of PON1 activity is higher than that of HDLC (Navab et al. 1997).

In accordance with our results, a recent study reported that the clinical significance of HDLC concentrations in the general population is markedly heterogeneous: high concentrations do not necessarily lower the cardiometabolic risk, putting the beneficial role of HDLC in doubt (Voight et al. 2012).

Although few previous in vitro or animal studies have shown antioxidant properties and the oxidative stress modulatory potential of clozapine (Dalla Libera et al. 1998; Pillai et al. 2007), we found that this atypical antipsychotic drug reduced TEAC and GAP, suggesting that it has a negative impact on serum antioxidant protection in human subjects. To our knowledge, this is the first study to evaluate the impact of long-term clozapine and risperidone treatment on TEAC and GAP in human subjects with schizophrenia.

Decreased PON(DHC) activity in our study might be responsible for the observed reduction in TEAC and GAP. Although clozapine showed lower levels of oxidative stress (measured as plasma malondialdehyde) than risperidone after 3 weeks of treatment in a previous study (Stefan Kropp et al. 2005), our results suggest that long-term clozapine treatment has a stronger negative impact than risperidone on the plasma antioxidant protection and on the anti-atherogenic capacity of the HDL particles. This pattern also recalls the dyslipidemic potency pattern of these drugs (Allison et al. 1999b; Baptista et al. 2008).

In conclusion, clozapine or risperidone treatment of patients with schizophrenia affects various paraoxonase activities, reducing PON(DHC) and increasing PON, PON(NaCl), and PON(PA). The decrease in PON(DHC) activity was concomitant with a paradoxical increase in HDLC in our subjects with schizophrenia. Based on our results, we suggest that the paraoxonase/HDLC ratio may be a better candidate for a cardiovascular risk marker than HDLC or paraoxonase activity alone.

The influence of clozapine on all the measured parameters was stronger than that of risperidone, showing that it could have a stronger negative impact on cardiovascular risk. The results of the present study also suggest that patients with schizophrenia might be at increased risk for metabolic and cardiovascular disease related to reduced PON(DHC), TEAC, and GAP.

We found no sex influence on the levels of all the measured parameters. The topic of gender differences in antioxidant status is complex, not sufficiently studied, and sometimes controversial. There are studies that found no gender differences (e.g., TEAC) (Rahman et al. 2000), while others reported such sex dependence of various parameters (e.g., TEAC, PON) (Valabhji et al. 2001; Winnier et al. 2007).

Because paraoxonase and plasma antioxidants can be modulated by diet (Esfahani et al. 2011; Koncsos et al. 2011; Rantala et al. 2002), our results indicate that patients with schizophrenia under long-term clozapine and risperidone treatment might benefit from dietary interventions. However, further prospective studies will be requested to test this hypothesis. We also suggest that patients who receive clozapine or risperidone treatment should be followed up more carefully in terms of cardiovascular risk.

There are a number of limitations to this study. The patient sample included individuals with schizophrenia under chronic (longer than 1 year) atypical antipsychotic drug treatment. The results may not be generalizable to patients with shorter treatment, other psychotic disorders, or those receiving antipsychotic medication for other illnesses. Due to the cross-sectional design of the study, it is difficult to make causal inference. Also the results should be interpreted with caution, since they may differ when another time frame would be chosen. Future prospective studies should confirm our conclusions.

The significant differences in BMI and age between the groups represent other limitations, despite the fact that they were considered confounding factors and regression analysis was used. Another limitation is that several aspects (e.g., exercise habits) known to influence oxidative parameters were not evaluated.

This study only dealt with two atypical antipsychotic medications. Further research is required to determine how the findings of this study are relevant to treatment with other antipsychotic medications, and the results presented here should be interpreted with caution. Although our study had a limited sample size, this could be advantageous in that it is more likely to have biased the results toward a negative finding because of insufficient statistical power.

## Abbreviations

PON: Paraoxonase
PON(PO): Paraoxonase’s activity against paraoxon
PON(DHC): Paraoxonase’s activity against dihydrocoumarin
PON(PA): Paraoxonase’s activity against phenylacetate
HDLC: HDL-cholesterol
TEAC: Trolox equivalent antioxidant activity
GAP: Antioxidant gap
